# Clinical Insights and Potential Benefits of Stem Cell Transplantation for Constitutional Mismatch Repair Deficiency: A Case Report of Two Siblings

**DOI:** 10.7759/cureus.66441

**Published:** 2024-08-08

**Authors:** Miłosz Mandryk, Joanna Owoc-Lempach, Jakub Cecot, Konrad Zarzecki, Małgorzata Piasta, Magdalena Wolska-Kolmus, Paweł Marschollek, Monika Mielcarek-Siedziuk, Bożenna Dembowska-Bagińska, Krzysztof Kałwak

**Affiliations:** 1 Pediatric, Hematology, Oncology and BMT, Wroclaw Medical University, Wroclaw, POL; 2 Medicine, Jan Mikulicz-Radecki University Clinical Hospital, Wroclaw, POL; 3 Medicine, 4th Military Clinical Hospital, Wroclaw, POL; 4 Medicine, Voivodeship Hospital, Kielce, POL; 5 Oncology, The Children's Memorial Health Institute, Warsaw, POL

**Keywords:** constitutional mismatch repair deficiency, hematopoietic stem cell transplantation, café au lait macules, glioblastoma, t-cell mediastinal lymphoma

## Abstract

Constitutional mismatch repair deficiency (CMMRD) syndrome, caused by biallelic mutations in mismatch repair genes, is one of the most aggressive hereditary cancer syndromes. This report presents the clinical course of two brothers diagnosed with CMMRD. The first patient was diagnosed with T-cell lymphoma at the age of three and a half years, a relapse, and synchronous glioblastoma at the age of seven and a half years. After treatment with chemotherapy and neurosurgery, haematopoietic stem cell transplant (HSCT) was performed. The second patient was diagnosed with mediastinal T-cell lymphoma at the age of two and a half years and a relapse at the age of four and a half years. He also received chemotherapy and underwent HSCT. Both patients exhibited café au lait macules (CALMs), a common but non-specific feature of CMMRD, often confused with neurofibromatosis type 1 (NF1) syndrome. This study highlights the phenotype of CMMRD syndrome, associated cancers, and the potential benefits of stem cell transplantation. Previous reports suggest that allogeneic HSCT might reduce subsequent haematological malignancies and increase survival.

## Introduction

Constitutional mismatch repair deficiency (CMMRD) syndrome is caused by biallelic mutations in mismatch repair (MMR) genes. It is the most aggressive and complex among hereditary cancer syndromes, with a very high incidence of various types of malignancies, which lead to excessive morbidity and reduced life span of affected individuals. Biallelic mutations (homozygous or compound heterozygous) affect MMR genes, such as *MLH1*, *MSH2*, *MSH6*, and *PMS2 *[1].

Monoallelic mutations in MMR genes cause Lynch syndrome (LS), predisposing individuals mainly to colorectal cancer, endometrial cancer, and other malignancies. Parents with LS have a one-quarter chance of having a child with biallelic pathogenic variants in MMR genes, leading to CMMRD [2]. Patients with CMMRD develop LS-related carcinomas, malignant brain and central nervous system tumours, haematological malignancies, and other types of cancer [3].

The presence of café-au-lait macules is often reported, less commonly Lisch nodules, spotting in the armpits and groin, and even neurofibromas that may resemble those found in neurofibromatosis type 1 syndrome (NF1). The frequent occurrence of these features, combined with the extreme rarity of CMMRD, makes a timely diagnosis challenging [4,5].

By 2019, just over 200 cases of CMMRD had been described in the literature. Creation of international databases, such as the European database 'Care for CMMRD' (C4CMMRD) and the International Replication Repair Deficiency Consortium (IRRDC) database, have significantly expanded our knowledge. Nevertheless, due to its rarity and limited access to genetic testing in many countries, the syndrome remains underdiagnosed [6].

In this report, we present the case of two brothers with CMMRD syndrome.

## Case presentation

Patient 1

**Figure 1:**
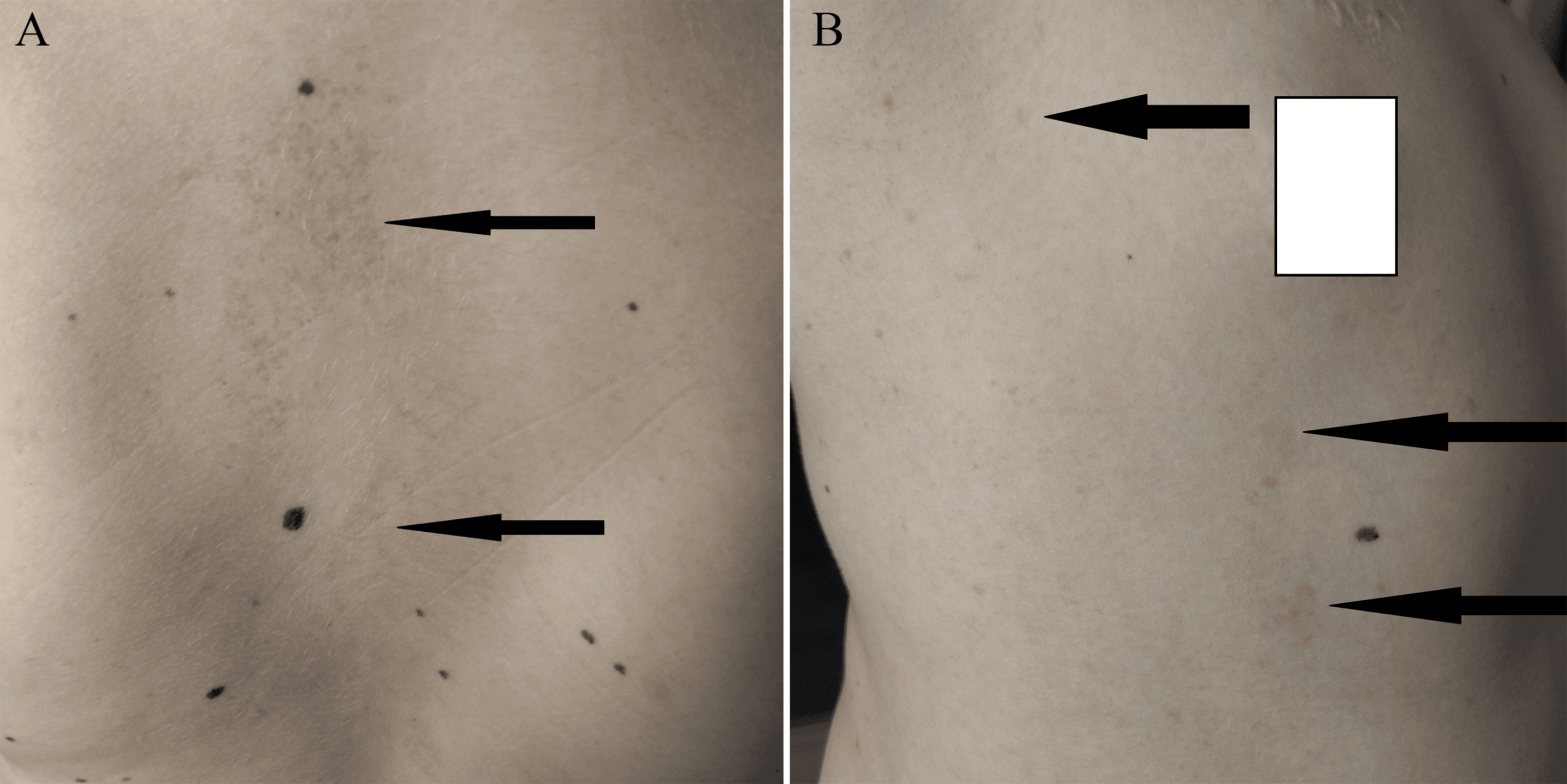
Café au lait spots on the skin of Patient 1 (photo shared with consent of parents) (A) Back skin area and (B) armpit skin area

A boy, now aged 11.5 years, born vaginally at 40 weeks of gestation, with a birth weight of 3,750 grams, scored 10 points on the Apgar scale. In infancy, his development was harmonious. At the age of three and a half years, the boy presented with symptoms of recurrent pneumonia despite appropriate therapy. In addition, dyspnea and facial swelling worsening in the supine position indicative of superior vena cava syndrome were observed. Only six months later, the presence of a mediastinal tumour was noted, for which thoracoscopy with lymph node retrieval was performed and confirmed the diagnosis of T-cell lymphoma. Treatment was carried out according to the EURO-LB 02 protocol. Remission of the disease was achieved and confirmed on imaging studies. Around the age of five years, the parents noticed the appearance of café au lait spots, which were mainly located in the groin, on the back (Figure 1A), and armpit area (Figure 1B).

At the age of six years - the time of completion of treatment - the patient’s younger brother was also diagnosed with mediastinal T-cell lymphoma. Detailed family history revealed that there were cases of children dying at a very young age among second-degree relatives in the family for reasons unknown. This finding raised suspicion of a genetic cancer predisposition syndrome. Genetic testing revealed the presence of a mutation in the *PMS2* gene, resulting in paired nucleotide mismatch repair deficiency syndrome.

**Figure 2:**
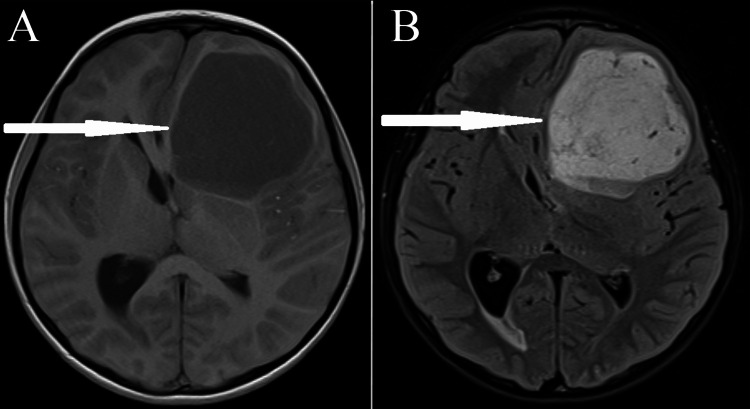
Images from the MRI scan: glioblastoma located in the left frontal lobe (A) T1 sequence and (B) T2 sequence

At the age of seven and a half, the patient complained of headaches, morning nausea, and vomiting. MRI of the head revealed a presence of a large left frontal lobe tumour, contrast-enhancing (Figure 2).

**Figure 3:**
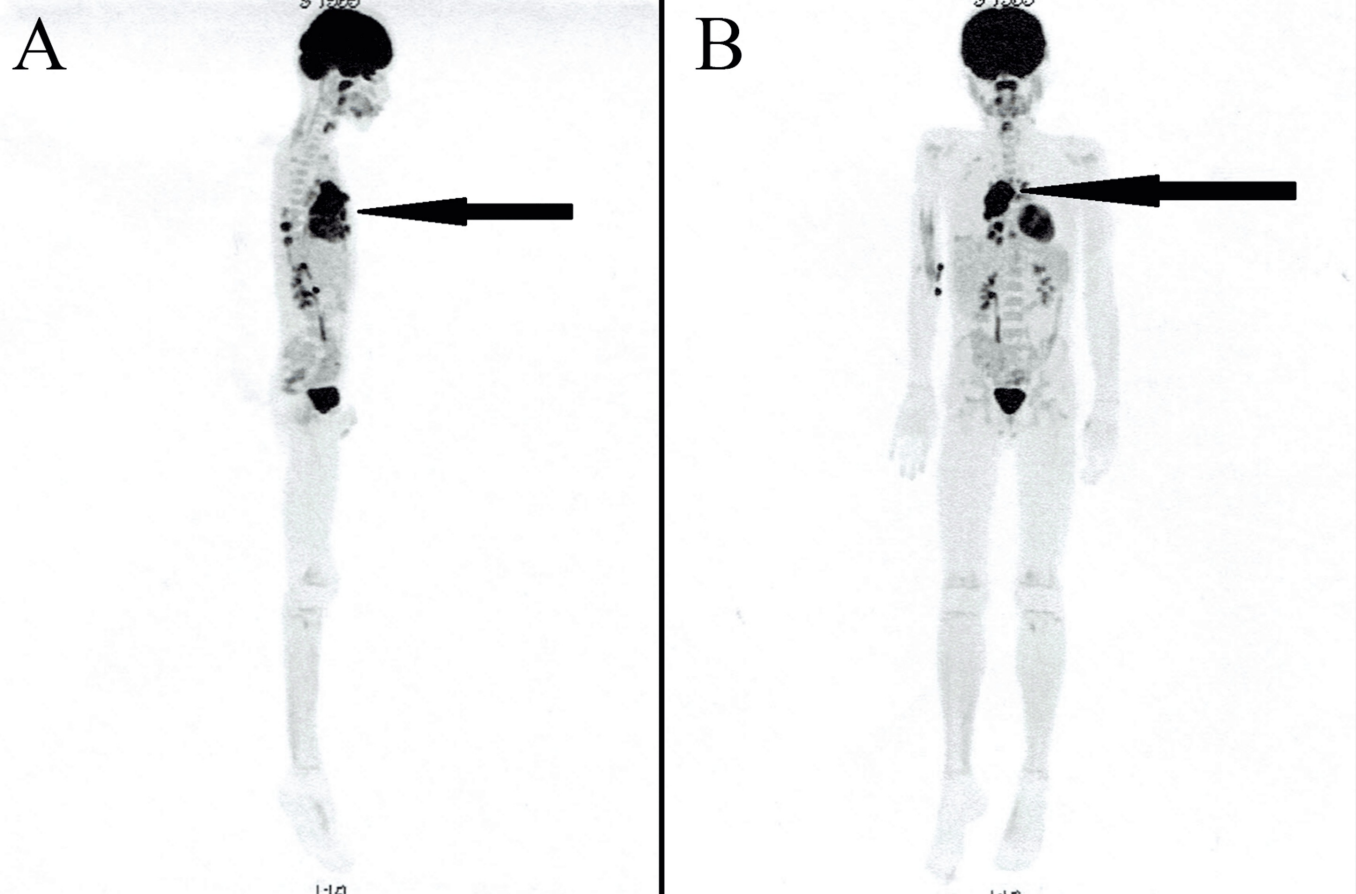
PET-CT scan images: T-cell lymphoma recurrence - tumour located in the mediastinum (A) Lateral view and (B) anterior view

The lesion was macroscopically completely excised. Histopathological examination confirmed glioblastoma. Subsequently, the boy was transferred to the Oncology Clinic for continuation of treatment, where an asymptomatic late isolated local recurrence of T-cell lymphoma was simultaneously diagnosed (Figure 3).

Initially, a first course of individual chemotherapy with VCR (vincristine), IF (ifosfamide), and ADM (adriamycin) was administered with concomitant steroid therapy. The patient was then treated with radiotherapy with photons and received a total dose of 4,500 cGy to the tumour locus area of the right frontal lobe using the volumetric modulated arc therapy (VMAT) technique. Subsequently, the patient started chemotherapy for T-cell lymphoma according to the IntReALL relapse protocol - standard-risk group. A follow-up CNS MRI revealed no signs of a frontal lobe tumour.

The patient was qualified for hematopoietic stem cell transplantation (HSCT) from a 9/10 human leukocyte antigen (HLA)-compatible unrelated donor (mismatch in DBQ1). Conditioning was performed with busulfan 0.8 mg/kg, fludarabine 5 x 30 mg/m^2^, thiotepa 2 x 5 mg/kg, and anti-thymocyte globulin (ATG) 45 mg/kg. Haematopoietic stem cells from peripheral blood were transplanted at a dose of 6.06 x 10^6^ CD34+ cells/kg of the recipient’s body mass. No early complications were observed. Haematological recovery was stimulated with G-CSF from day +7 after transplantation. Recovery in the leukocytes and granulocytes was achieved on day +12 and in platelets on day +14 after HSCT. The post-transplant period was complicated by diarrhoea probably due to intestinal graft-versus-host disease (grade II), successfully controlled with steroid therapy. No further serious complications were observed. The patient was discharged on day +51 post-transplant and has been followed at the transplant clinic since then.

Follow-Up

The patient has been in full remission from lymphoma for three years. Due to IVS cardiac hypokinesis, the patient is treated with enalapril. Additionally, for adrenal insufficiency, he was started on supplementation by hydrocortisone. A follow-up abdominal ultrasound scan raised the suspicion of focal nodular proliferation of the liver, which requires further diagnosis. Due to recurrent melena, a colonoscopy was performed, revealing multiple colonic polyps. Subsequent colonoscopies have consistently detected new polyps. A recent follow-up colonoscopy identified five colorectal polyps, four of which were removed. Histopathological examination was consistent with a low degree of dysplasia.

Patient 2

A boy, now aged seven and a half years, born vaginally at 40 weeks of gestation, with a birth weight of 3,580 grams, scored 10 points on the Apgar scale. In early infancy, his development was normal. At the age of about two and a half years, he developed a cough that worsened in the supine position, along with fevers that did not respond to antipyretics and antibiotics. Dilation of superficial chest vessels and dyspnea were observed.

**Figure 4:**
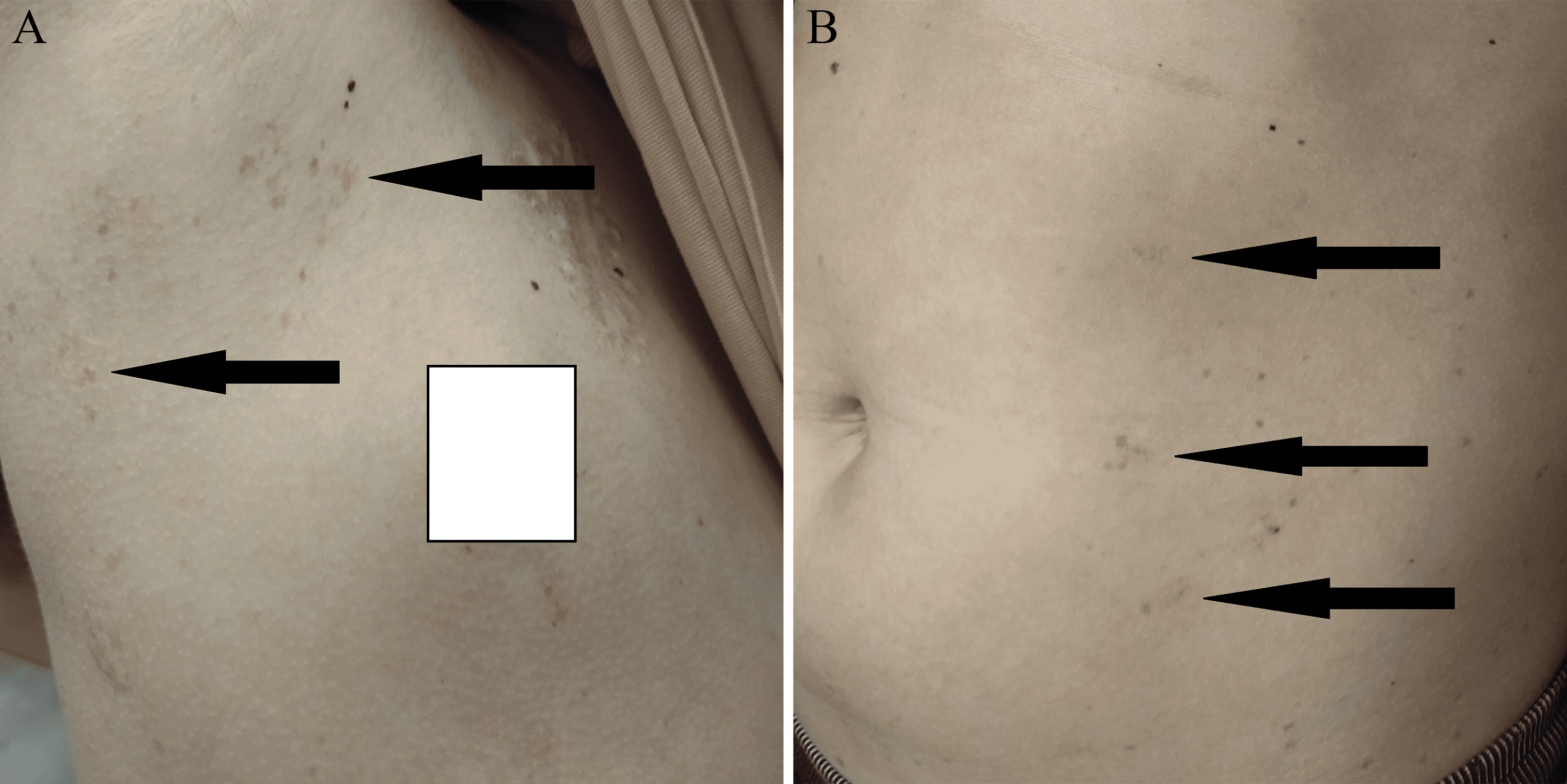
Café au lait spots on the skin of Patient 2 (photo shared with consent of parents) (A) Armpit skin area and (B) abdominal skin area

Within one and a half months of the first symptoms, a diagnosis was established - mediastinal T-cell lymphoma. Due to a suspicion of genetic cancer predisposition syndrome, a genetic test confirmed a mutation in the *PMS2* gene. The patient received chemotherapy according to the EURO-LB 02 protocol. He entered a complete remission and completed treatment at the age of three and a half years. At the same age, cafe au lait spots appeared on the boy's skin, and increased gradually over time, located mainly under the armpits (Figure 4A) and on the abdomen (Figure 4B).

One year later, a late isolated local recurrence of the disease was diagnosed, and chemotherapy was started according to the IntReALL SR protocol. Follow-up examinations confirmed complete remission of the disease. Subsequently, this patient was also qualified for a bone marrow stem cell transplant from an unrelated donor.

Conditioning was performed with total body irradiation, etoposide dosed at 60 mg/kg and anti-thymocyte globulin (ATG) dosed at 45 mg/kg. Haematopoietic stem cells from peripheral blood were transplanted at a dose of 7.92 x 10^6^ CD34+ cells/kg of the recipient. No early complications were observed. Granulocytes and leukocytes recovered on day +12. Platelet recovery was achieved on day +14 after HSCT. A cutaneous form of graft-versus-host disease (grade II) was diagnosed on day +14. Steroid therapy was administered with good results. Due to secondary hypothyroidism, the patient required replacement therapy. No further serious complications were observed. The patient was discharged on day +72 post-transplant.

Follow-Up

The patient has been in full remission from lymphoma for three years. Due to adrenal insufficiency, hydrocortisone has been prescribed. Two and a half years after transplantation, a new asymptomatic focal lesion in the left temporal lobe was spotted in an MRI scan of the brain and removed by temporal craniotomy. Histopathological examination revealed that it was a nonmalignant vascular malformation. As with the previous patient, each colonoscopy shows the presence of new colorectal polyps. Histopathological examination revealed a low degree of dysplasia in the samples taken.

## Discussion

We aim to emphasise the phenotype of CMMRD, the characteristics of the cancers that develop in these patients, and the potential benefits of haematopoietic stem cell transplantation in CMMRD syndrome patients.

A recent large cohort study presented data on patients with CMMRD syndrome collected over several years from more than 50 countries. This study included 201 patients with CMMRD, reporting a total of 339 cancers in 194 individuals. Approximately 51% (n=173) of the malignancies were central nervous system tumours, with glioblastoma being the most common, accounting for 66% (n=115) of all CNS tumours. These findings are reflected in our first patient who developed glioblastoma. Both of our patients developed haematological malignancies, which accounted for approximately 18% (n=61) of all malignancies in the study. Both were diagnosed with mediastinal T-cell lymphoma, representing 43% (n=26) of all haematological neoplasms. Genetically, mutations in the *PMS2* gene occur in the majority of CMMRD patients, accounting for approximately 65% of all mutations, our patients included [7].

Haematological cancers were common among *PMS2* or *MSH6* mutation carriers, while gastrointestinal and CNS cancers were prevalent across all four MMR genes. Patients with CNS tumours and *PMS2* or *MSH6* mutations had significantly better five-year overall survival rates compared to those with *MLH1* or *MSH2* mutations [7].

Our patients exhibited café au lait macules (CALMs) that first appeared at five and three and a half years of age, respectively. They are observed in approximately 89% of CMMRD cases, often initially suggesting NF1 syndrome. Making the diagnosis even more challenging, about 25% of CMMRD patients present multiple features of NF1 and meet the clinical criteria of the National Institutes of Health (NIH) but lack germline pathogenic variants in *NF1* or *SPRED1* [7,8]. CALMs in CMMRD patients can differ in pigmentation and may have irregular shapes compared to those in NF1 syndrome, making them distinguishable only by very experienced clinicians [9].

In the literature, there are several cases of patients with CMMRD syndrome who underwent HSCT [10]. Given the limited knowledge in this area, it is difficult to draw a conclusion about the real benefits of this procedure for these patients. From the clinical case reports and observations published to date, it appears that allogeneic HSCT might reduce the risk of subsequent haematological malignancies and therefore increase survival among these patients [11-13].

Ripperger et al. [10,11] reported a patient who underwent chemotherapy for mediastinal T-cell lymphoblastic lymphoma (T-NHL) at the age of six years. The patient was later diagnosed with CMMRD syndrome (mutation in the *MSH6* gene). The patient was in complete remission but relapsed shortly after completion of treatment. Since allogeneic hematopoietic stem cell transplantation (allo-HSCT) was performed, he remains in second complete remission. At the age of 13 years, a colonoscopy was performed due to lower gastrointestinal bleeding. Disturbing lesions indicative of cancer were detected, which were spread throughout the large intestine. A colectomy with a terminal ileostomy was performed. Histopathological examination revealed bifocal adenocarcinoma of the colon with metastasis to local lymph nodes. One year later, rectal adenocarcinoma with low-grade intraepithelial atypia was detected. A proctectomy with endoanal mucosectomy and anastomosis of the ileum to the rectum was performed. The patient remains under surveillance, with no signs of T-NHL relapse at the time of reporting [11]. In 2018, Leenders et al. described a case of an eight-year-old patient with CMMRD syndrome (mutation in the *PMS2* gene). The patient suffered a relapse of T-NHL isolated to the testicles. He received reinduction chemotherapy, followed by an allo-HSCT from an HLA-identical sibling. The patient was alive and well at the time of reporting, almost four years in second complete remission following the transplant [12].

In a different cohort study on T-NHL, a total of 88 paediatric patients were considered, eight of whom were diagnosed with CMMRD syndrome. One of these patients (with a mutation in the *PMS2* gene) underwent allo-HSCT, following a relapse during maintenance treatment. With this management, the patient was rescued. At the time of reporting, he was in complete remission for seven years after transplantation [13].

In the case of the patients we presented, they remain in complete remission for over three years and are followed closely in a transplant clinic. 

## Conclusions

In conclusion, based on the example of the siblings that we presented in this paper, HSCT can be carried out in CMMRD patients without serious complications. However, there may be a limited advantage as it probably does not affect the incidence of other non-haematological malignancies. It is crucial to continue a long-term follow-up and collect more data on patients with CMMRD syndrome who have undergone allo-HSCT to better understand its long-term effects. This may contribute to a longer survival and lower morbidity in CMMRD patients.
